# Development of a poor-prognostic-mutations derived immune prognostic model for acute myeloid leukemia

**DOI:** 10.1038/s41598-021-84190-0

**Published:** 2021-03-01

**Authors:** Feng-Ting Dao, Jun Wang, Lu Yang, Ya-Zhen Qin

**Affiliations:** Peking University People’s Hospital, Peking University Institute of Hematology, National Clinical Research Center for Hematologic Disease, No. 11 Xizhimen South Street, Xicheng District, Beijing, 100044 China

**Keywords:** Haematological cancer, Leukaemia, Acute myeloid leukaemia, Cancer, Immunology

## Abstract

Leukemia cell-intrinsic somatic mutations and cytogenetic abnormalities have been used to define risk categories in acute myeloid leukemia (AML). In addition, since the immune microenvironment might influence prognosis and somatic mutations have been demonstrated to modulate the immune microenvironment in AML, there is need for developing and evaluating an immune prognostic model (IPM) derived from mutations associated with poor prognosis. Based on AML cases with intermediate and adverse-cytogenetic risk in the Cancer Genome Atlas (TCGA) database, 64 immune-related differentially expressed genes (DEGs) among patients with *RUNX1*, *TP53*, or *ASXL1* mutations and patients without these mutations were identified. After Cox proportional hazards analysis, an IPM composed of *PYCARD* and *PEAR1* genes was constructed. IPM defined high-risk (IPM-HR) independently predicted lower 2-year overall survival (OS) rates in both patients with intermediate and adverse-cytogenetic risks and non-M3 patients in the TCGA AML cohort. The poor prognostic impact of IPM-HR on OS was further validated by GSE71014, 37642, and 10358 downloaded from the Gene Expression Omnibus (GEO) database. Furthermore, IPM-HR was remarkably associated with higher proportions of CD8+ T cells and regulatory T cells (Tregs), lower proportions of eosinophils, and higher expression of the checkpoint molecules *CTLA-4*, *PD-1*, and *LAG3* in the TCGA non-M3 AML cohort. In summary, we developed and validated an IPM derived from mutations related with poor prognosis in AML, which would provide new biomarkers for patient stratification and personalized immunotherapy.

## Introduction

Cytogenetic abnormalities are the backbone of risk stratification in acute myeloid leukemia (AML), a malignant hematological disease, and more than 2/3 of patients with AML are classified as intermediate and adverse-cytogenetic risk groups^1–3^. Over the past two decades, several somatic mutations have been proven to be strongly prognostic in AML and have been incorporated into risk categories in both NCCN guidelines and ELN recommendations^4–8^. For example, AML patients with unfavorable cytogenetic risk harboring *RUNX1*, *TP53*, or *ASXL1* mutations are defined as adverse risk categories.

In addition to the leukemia cell-intrinsic mechanism, the immune microenvironment plays an important role in the pathogenesis of AML and might influence the prognosis of patients with this disease^9–11^. Furthermore, some leukemia-related somatic mutations have been demonstrated to modulate the immune microenvironment in AML^12–18^. For instance, the *RUNX1* mutation has been demonstrated to modulate nuclear factor (NF)-kB signaling and promote inflammatory signaling in the bone marrow microenvironment^12^. *TP53* activates transcription of critical regulators of the innate immune response, and dysregulation of pathways downstream of mutated *TP53* may mediate resistance to chemotherapy^13–16^. *ASXL1* plays an important role in the microenvironment to support normal hematopoiesis, and mutant *ASXL1* proteins gain functions that promote myeloid leukemogenesis^17,18^. Therefore, we speculate that the poor prognosis of patients with AML harboring *RUNX1*, *TP53*, or *ASXL1* mutations may be partly caused by the specific influences of these mutations on the leukemia-associated immune system.

At present, the immune cell-specific gene expression profiles have been clarified. Based on multiple gene expression data and their association with treatment outcomes, the immune prognostic model (IPM) has been widely used to stratify patients with solid tumors, such as colorectal cancer^19^, hepatocellular carcinoma^20,21^, and lung cancer^22^. Nevertheless, no study has established an IPM in AML so far.

In the present study, we downloaded gene expression data of AML cohorts from The Cancer Genome Atlas (TCGA) database, identified the expression of *RUNX1*, *TP53*, and *ASXL1* mutation-associated genes, and established an IPM. Furthermore, we demonstrated that the IPM-defined risk strongly predicted overall survival (OS) in the TCGA AML cohort and this was validated by three GEO datasets.

## Results

### *RUNX1*,* TP53*, and *ASXL1* mutations associated with immune profile in AML with intermediate and adverse-cytogenetic risk in the TCGA cohort

**Figure 1:**
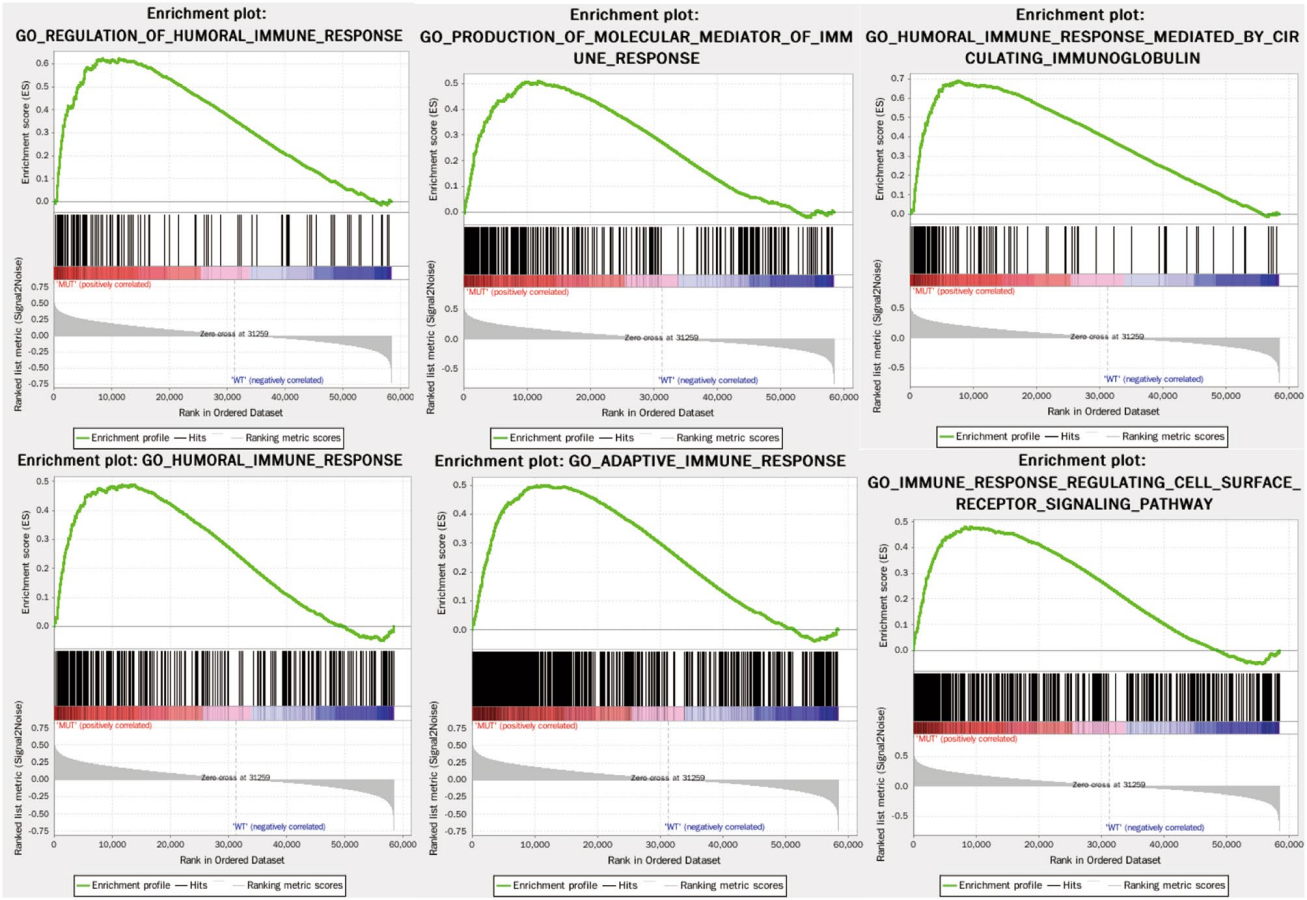
Comparison between significantly enriched immune-related phenotypes in the patients of the MUT and WT groups based on GSEA analysis.

According to NCCN and ELN guidelines, non-favorable cytogenetic risk AML patients with *RUNX1*, *TP53*, or *ASXL1* mutations were defined as adverse risk categories. Thus, we adopted AML patients with intermediate and adverse-cytogenetic risk to explore the *RUNX1*, *TP53*, and *ASXL1* mutations associated with the immune signature. Of 173 AML patients in TCGA database, a total of 116 patients with available gene expression profile and mutation status had intermediate and adverse-cytogenetic risk and were analyzed. The *RUNX1*, *TP53*, and *ASXL1* mutation frequencies were 12.1% (14/116), 9.5% (11/116), and 1.7% (2/116), respectively. A total of 25 patients had at least one somatic mutation (*RUNX1*, *TP53*, or *ASXL1*) and were categorized as the MUT group, and the remaining 91 patients had no mutations in *RUNX1*, *TP53*, and *ASXL1* and were categorized as the WT group. GSEA analysis of the MUT and WT groups showed that the MUT group genetic profiles were significantly enriched in 445 biological processes, and six immune-related biological processes were included: REGULATION_OF_HUMORAL_IMMUNE_RESPONSE (NES = 1.80, size = 128, *P* = 0.031), PRODUCTION_OF_MOLECULAR_MEDIATOR_OF_IMMUNE_RESPONSE (normalized enrichment score, NES = 1.79, size = 272, *P* = 0.014), HUMORAL_IMMUNE_RESPONSE_MEDIATED_BY_CIRCULATING_IMMUNOGLOBULIN (NES = 1.77, size = 137, *P* = 0.036), HUMORAL_IMMUNE_RESPONSE (NES = 1.75, size = 337, *P* = 0.032), ADAPTIVE_IMMUNE_RESPONSE (NES = 1.67, size = 603, *P* = 0.033), and IMMUNE_RESPONSE_REGULATING_CELL_SURFACE_RECEPTOR_SIGNALING_PATHWAY (NES = 1.62, size = 482, *P* = 0.032) (Fig. 1). Thus, 1958 immune-related genes were obtained from these six immune-related processes.

### Differentially expressed immune-related genes between the MUT and WT groups

In order to identify more candidate immune-related DEGs, two different methods were used. Firstly, 897 DEGs (822 upregulated and 75 downregulated) were identified between the MUT and WT groups (|log2FC|≥ 1.0 and FDR < 0.05), and 25 of them were shown to be involved in immune-related biological processes (FDR < 0.05) by gene ontology (GO) enrichment analysis. Secondly, of the 1,958 immune-related genes obtained from GSEA analysis, 51 genes were found to be differentially expressed between the MUT and WT groups (|log2FC|≥ 1.0 and FDR < 0.05).Finally, excluding 12 repetitive DEGs, 64 DEGs in total between MUT and WT groups were shown to be immune-related and used for the design of the IPM.

### Establishment of an IPM and evaluation of its predictive ability

Of 116 AML patients with intermediate and adverse-cytogenetic risk in TCGA database, 107 patients had survival information and survival analysis was performed. Their median follow-up period was 305 days (range: 0–2861 days) and the 2-year OS rate was 37.7% (95% confidence interval [CI]: 27.5–47.7%).

**Figure 2:**
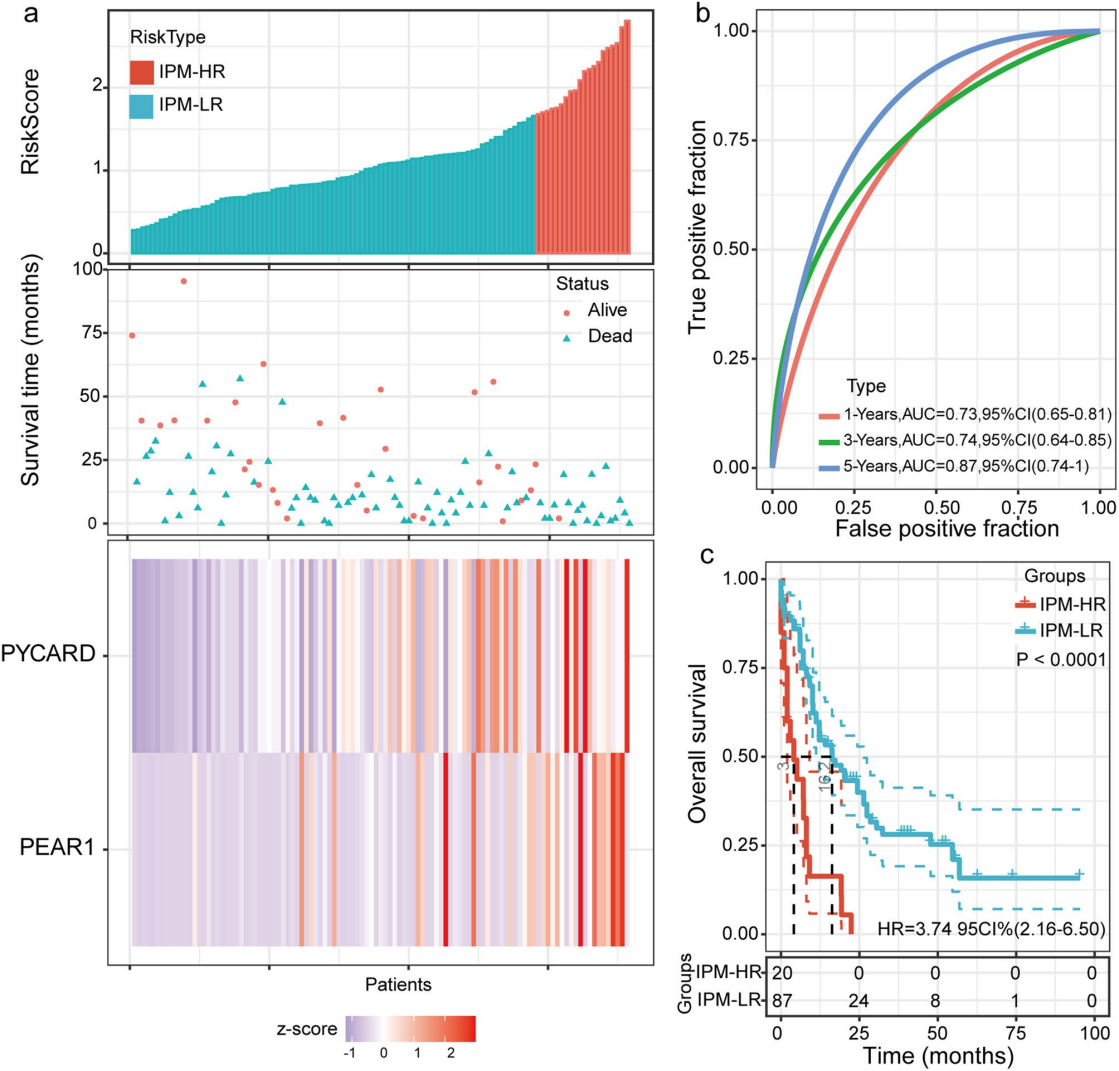
Establishment of an IPM and evaluation of its predictive ability in TCGA AML patients with intermediate and adverse-cytogenetic risk. (**a**) Risk score distribution and the corresponding survival and gene expression data; (**b**) Time-dependent ROC curve of the IPM; (**c**) Comparison between overall survival in the IPM-HR and IPM-LR groups.

Univariate Cox regression analysis revealed that three of the 64 immune-related DEGs were significantly related to OS (Table S1). However, only two genes, PYD and CARD domain-containing (*PYCARD*) and platelet endothelial aggregation receptor 1 (*PEAR1*), were shown to be significantly prognostic (i.e.*, P* < 0.05) in multivariate Cox regression analysis (both were poor prognostic factors). Then, an IPM based on these two genes was established by Cox proportional hazards analysis, and a risk score to predict prognostic value was calculated. The median IPM defined risk score for AML patients with intermediate and adverse-cytogenetic risk in the TCGA cohort was 1.07 (range: 0.28–2.81), and the risk score distribution and gene expression data are shown in Fig. 2a. As shown in Fig. 2b, the AUC of the IPM for OS was 0.73 at one year, 0.74 at three years, and 0.87 at five years.

The ROC curves showed that 1.67 had a maximum Youden index (0.22) among all values. Thus, 1.67 was identified as the optimal cutoff value of the IPM defined risk score, risk score ≥ 1.67 and < 1.67 were defined as the IPM-HR and IPM-LR groups, respectively. In AML patients with intermediate and adverse-cytogenetic risk, 20 (18.7%) and 87 (81.3%) patients were individually categorized into the IPM-HR and IPM-LR groups, respectively. As a result, patients in the IPM-HR group had a significantly lower 2-year OS rate than the patients in the IPM-LR group (0 vs. 45.9% [95% CI: 34.1–56.9%], *P* < 0.0001, Fig. 2c; Table S2).

### Relationship between IPM-defined risk and patient characteristics in the TCGA cohort

As shown in Table S3, of 107 AML patients with intermediate and adverse-cytogenetic risk, IPM-HR was significantly related to *RUNX1* mutation (*P* = 0.023) and advanced age (*P* < 0.0001). However, the IPM defined risk had no relationship with sex, white blood cell (WBC) counts, hemoglobin (HB) levels, platelet (PLT) counts, percentage of bone marrow (BM) blast, FAB subtype, *ASXL1* mutation, *TP53* mutation, *FLT3-ITD* mutation, *NPM1* mutation, *CEBPA* biallelic mutation, *WT1* mutation*,* and *DNMT3A* mutation (all *P* > 0.05).

### IPM-defined risk independently predicted poor outcomes in the TCGA cohort

As shown in Table S2, in AML patients with intermediate and adverse-cytogenetic risk, older age, *TP53* mutation, and *DNMT3A* mutation were all significantly related to a lower 2-year OS rate in addition to IPM-HR (all *P* < 0.05). Multivariable analysis showed that IPM-HR, older age, *TP53* mutation, *FLT3-ITD*, and *DNMT3A* mutations were all independent adverse prognostic factors for OS in AML patients with intermediate and adverse-cytogenetic risk (Table 1).

**Table 1:** Independent prognostic factors for OS of AML patients in TCGA cohort.

	AML patients with intermediate and adverse-cytogenetic risk			Non-M3 AML patients		
	No. of patients	HR(95%CI)	*P* value	No. of patients	HR(95%CI)	*P* value
**IPM**						
IPM-LR	87	1.00	< 0.0001	104	1.00	< 0.0001
IPM-HR	20	3.22 (1.71–6.05)		22	3.40 (1.84–6.27)	
**Age**						
Age < 60y	52	1.00	0.040	60	1.00	0.030
Age ≥ 60y	55	1.71 (1.03–2.85)		66	1.72 (1.06–2.81)	
**TP53**						
Wild type	97	1.00	0.001	114	1.00	< 0.0001
Mutation	10	4.05 (1.82–9.0)		10	4.26 (2.0–9.06)	
**FLT3-ITD**						
( −)	86	1.00	0.033	99	1.00	0.027
( +)	21	1.94 (1.06–3.58)		25	1.95 (1.08–3.52)	
**DNMT3A**						
Wild type	74	1.00	0.006	90	1.00	0.006
Mutation	33	2.07 (1.23–3.48)		34	2.08 (1.24–3.50)	

We further evaluated the prognostic impact of IPM risk score on OS in TCGA non-M3 AML patients. Of 126 patients with survival information, 22 (17.5%) and 104 (82.5%) patients were individually categorized into the IPM-HR and IPM-LR groups by a cutoff value of 1.67, and the IPM-HR group had a significantly lower 2-year OS rate than the IPM-LR group (0 vs. 49.6% [95% CI: 38.5–59.8%], *P* < 0.0001). Univariate analysis showed that IPM-HR, older age, *TP53* mutation, *DNMT3A* mutation, and intermediate and adverse-cytogenetic risk were all significantly related to a lower 2-year OS rate (all *P* < 0.05, Table S4). Similar to the results of patients with intermediate and adverse-cytogenetic risk, IPM-HR, older age, *TP53* mutation, *FLT3-ITD*, and *DNMT3A* mutation were all independent adverse prognostic factors for OS in non-M3 AML patients (Table 1).

### Validation of the prognostic impact of IPM-defined risk in the GEO database

**Figure 3:**
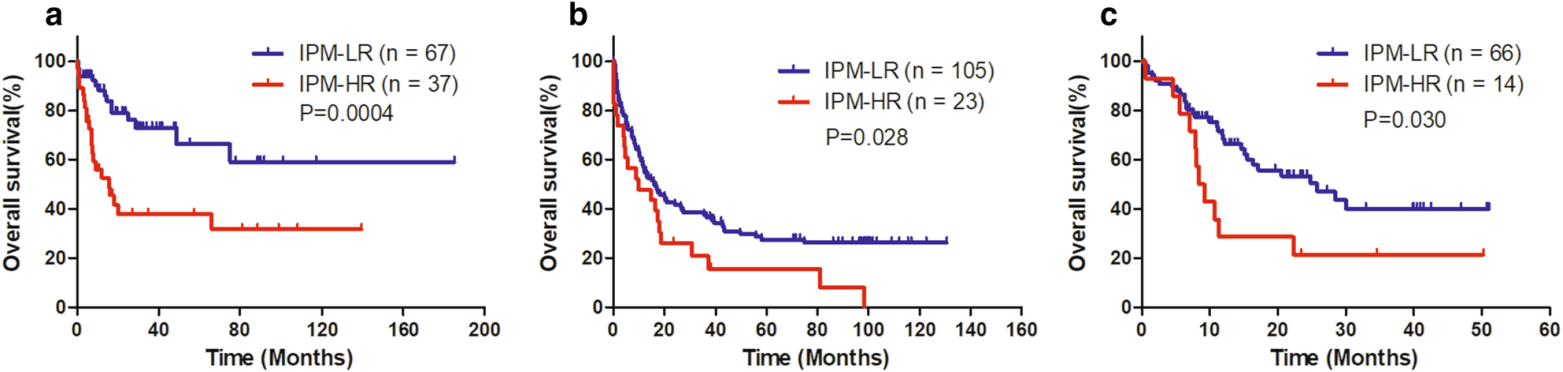
Validation of the prognostic impact of IPM defined risk on OS in three GEO datasets (**a**) OS of AML cases with normal karyotype from GSE71014 (**b**) OS of non-M3 AML cases from GSE37642 (**c**) OS of non-M3 AML cases from GSE10358.

A total of 104 AML cases with normal karyotype from GSE71014, 128 non-M3 AML cases from GSE37642, and 80 non-M3 AML cases from GSE10358 were used to verify the prognostic significance of the poor prognostic mutation-derived IPM defined risk. Patients in each cohort had their IPM risk score calculated and were divided into the IPM-HR and IPM-LR groups based on the individual ROC curve determined cutoff values. Consistent with the results in TCGA cohort, patients in the IPM-HR group had significantly lower 2-year OS rates than those in the IPM-LR group (GSE71014: 37.9 [95% CI:21.1–54.7%] vs 79.1% [95% CI: 65.0–88.0%], *P* = 0.0004, Fig. 3a; GSE37642: 26.1% [95% CI: 10.6–44.7%] vs 42.5% [95% CI: 32.8–51.8%], *P* = 0.028, Fig. 3b; GSE10358: 21.4% [95% CI: 5.2–44.8%] vs 53.4% [95% CI: 39.3–65.6%], *P* = 0.030, Fig. 3c).

### Immune cell infiltration landscapes and checkpoint molecule analysis of IPM-HR and IPM-LR patients

**Figure 4:**
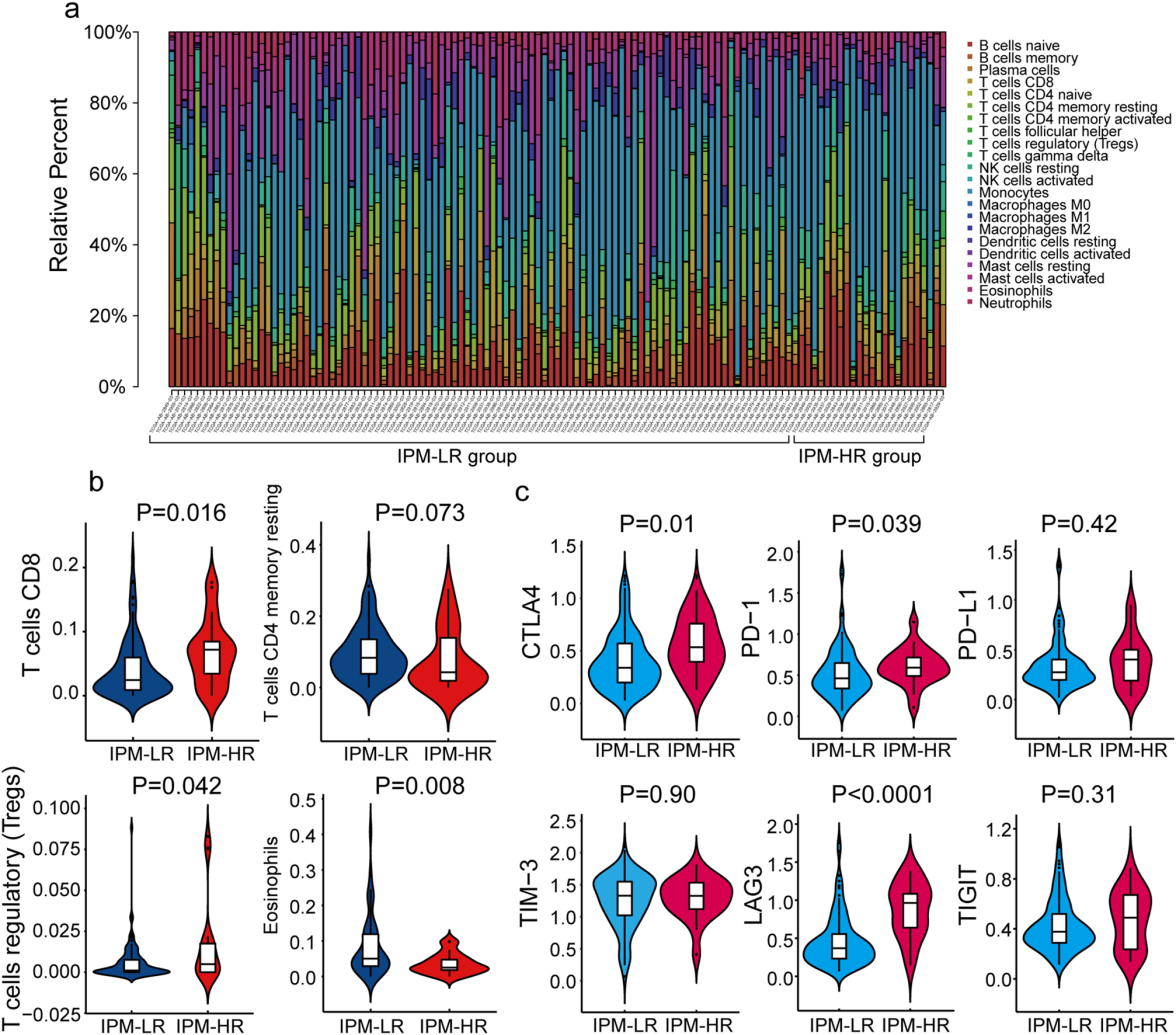
Analysis of the immune cells infiltration landscapes and checkpoint molecules between IPM-HR and IPM-LR groups in non-M3 AML patients from the TCGA cohort: (**a**) Relative proportion of immune cells infiltration in IPM-HR and IPM-LR patients; (**b**) Violin plots showing significantly different proportions of immune cells between IPM-HR and IPM-LR patients; (**c**) Violin plots showing the expression of different immune checkpoint molecules between IPM-HR and IPM-LR patients.

For 126 non-M3 AML patients in TCGA cohort, the proportions of 22 immune cell types were estimated using the CIBERSORT method and compared between the IPM-HR and IPM-LR groups (Fig. 4a). Patients in the IPM-HR group had significantly higher proportions of CD8 T cells and regulatory T cells (Tregs), lower proportions of eosinophils, and tended to have significantly lower proportions of resting CD4 memory T cells compared with patients in the IPM-LR group (*P* = 0.016, 0.042, 0.008, and 0.073, Fig. 4b).

Then, we compared the expression of critical immune checkpoint molecules (*CTLA-4*, *PD-1*, *PD-L1*, *TIM-3*, *LAG-3*, and *TIGIT*) between the IPM-HR and IPM-LR groups. The expression of *CTLA-4*, *PD-1*, and *LAG3* in the IPM-HR group was significantly higher than that in the IPM-LR group in non-M3 AML patients in TCGA cohort (*P* = 0.010, 0.039, and < 0.0001, Fig. 4c).

## Discussion

In the present study, we developed an *RUNX1*, *TP53*, and *ASXL1* mutation derived IPM based on TCGA AML cohort, and demonstrated that the IPM-defined risk independently predicted OS in AML, which was validated by GSE databases.

The first step in establishing an IPM is the identification of candidate genes. The somatic mutations associated with adverse risk included *RUNX1*, *TP53*, *ASXL1*, and *FLT3-ITD* in both ELN recommendations and NCCN guidelines. Because the prognostic role of *FLT3-ITD* is related to both the *NPM1* mutation status and *FLT3-ITD* mutation load, and the mutation load information was unavailable in TCGA database, only *RUNX1*, *TP53*, and *ASXL1* mutation status were considered for IPM development in the present study. For the comparison between poor-prognostic and non-poor prognostic groups, we identified DEGs in two ways in order to select as many candidates as possible, that is, immune-related genes in both GSEA and GO analysis. After univariate and multivariate Cox regression analyses, we established an IPM composed of two gene expression profiles with individual weights. Then, the cutoff value was determined by ROC curve analysis and used to group patients into the IPM-HR and -LR groups in each cohort, respectively. The survival analysis demonstrated that IPM defined high risk as an independent poor prognostic factor in both intermediate and adverse-cytogenetic risk AML patients and non-M3 AML patients in TCGA cohorts. Furthermore, this impact was individually validated in one cohort of AML with normal karyotype and two cohorts of non-M3 AML, which were downloaded from GEO databases. These results illustrate the usefulness of our IPM and also reflect that the immune microenvironment is involved in the prognosis of AML.

The IPM we developed was composed of *PYCARD* and *PEAR1* genes. *PYCARD* is a 22-kD protein containing an N-terminal pyrin domain (PYD) and a C-terminal caspase activation and recruitment domain (CARD)^26,27^. *PYCARD* expression is found in tumor cells, tumor-associated macrophages, normal epithelial cells, and non-tumor adjacent tissues^26–31^. It is silenced by methylation in many tumors, preventing tumor cells from apoptosis, which supports its role as a tumor suppressor^28–31^. Subsequent studies found that *PYCARD* is a central adaptor molecule of the inflammasome complex, which mediates the secretion of inflammatory cytokines (i.e., IL-1β and IL-18)^27,32^. Inflammation contributes to tumor development and progression, and inflammatory cytokines contribute to tumor promotion^33^. Therefore, in the context of cancer development and progression, *PYCARD* may exert opposing functions, either tumor-suppressing by inducing apoptosis or tumor-promoting by secretion of inflammatory cytokines within the tumor microenvironment. Contradictory results exist in the clinical relevance of *PYCARD* expression in various solid tumors^34–36^. *PEAR1* is a transmembrane protein that is mainly expressed on platelets, hematopoietic stem cells, and endothelial cells. It sustains αIIbβ3 activation in aggregating platelets and attenuates megakaryopoiesis by controlling the degree of Akt phosphorylation^37,38^. Moreover, methylation-controlled *PEAR1* expression can activate platelets and have an impact on inflammation^39–41^. Both in vitro and in vivo studies in endothelial cells showed an inverse correlation between *PEAR1* expression and vascular assembly, which implies that *PEAR1* modifies neo-angiogenesis^42^. Angiogenesis is a hallmark of cancer, and acute leukemia has been demonstrated to have increased angiogenesis, as assessed by microvessel density, compared to normal controls^43^. In addition, *PEAR1* regulates the early stages of hematopoietic differentiation^44^. Both expressions of *PYCARD* and *PEAR1* were poor prognostic indicators in our developed and validated IPM, whereas none of them has ever been reported in AML to date. Considering the published studies, we suspected that these may represent distinct mechanisms of the effect of microenvironment on AML, and mass cytometry or single cell sequencing followed by functional studies would be the way to clarify this.

Some studies hypothesized that during tumor development in immune-competent hosts, less immunogenic cancer cells are selected and immunosuppressive networks are established to evade antitumor immune responses^45,46^. Decreasing the expression of cancer antigens and immunoreactive cells such as follicular helper T cells, and increasing immunosuppressive molecules and cells such as Treg cells and tumor-associated macrophages are immunosuppressive mechanisms of cancers^47,48^. In this study, we found that patients in the IPM-HR group had higher proportions of CD8+ T cells and Tregs but lower fractions of eosinophils and CD4+ memory resting T cells than the IPM-LR group in non-M3 AML patients in the TCGA cohort*.* Then, we investigated the expression of immune checkpoints between the IPM-HR and IPM-LR groups. The IPM-HR patients had significantly higher expression of CTLA-4, PD-1, and LAG3 than the IPM-LR patients. CTLA4 and PD-1 have been demonstrated to play important roles in hampering T cell immunity against hematological malignancies and immune evasion of AML^49–52^. Antibodies targeting both the PD-1 and CTLA-4 pathways have shown efficacy in a variety of solid tumors and in some hematologic malignancies in clinical trials^33,53–55^. CD4+ T helper (Th) cells expressing upregulated PD-1 and/or LAG3 were identified together with CD86+ and/or ICOS-LG + myeloid blasts in the bone marrow of patients with AML, which may induce Th cell exhaustion and limit antitumor immunity^56^. Cytotoxic T lymphocytes (CTLs) are CD8+ T cells that play an important role in antitumor immune responses, but CD8+ T cells in the bone marrow of AML patients had reduced killing capacity and higher levels of PD-1 expressed^57^. CD4+ T cells can differentiate into Tregs, which are important in immunosuppressive mechanisms that lead to the inhibition of proliferation and cytokine production of other T cells^58^. Increased numbers of Tregs in solid tumors have been associated with worse outcomes and lead to the suppression of antitumor immunity^59^. Our results suggest that IPM-HR patients have higher expression of some immune checkpoint molecules and more Tregs forming an immunosuppressive environment, which may lead to a poor prognosis. Thus, IPM-HR patients may benefit from immunotherapeutic strategies targeting Tregs and immune checkpoint inhibitors.

Overall, we established and validated an IPM based on two immune microenvironment-related genes associated with *RUNX1*, *TP53*, or *ASXL1* mutation status, which independently predicted the overall survival of AML patients. IPM-HR was related to a higher proportion of Tregs and higher expression of checkpoint molecules CTLA-4, PD-1, and LAG3. The current IPM may provide a new biomarker for stratification and immunotherapeutic strategies for AML.

## Materials and methods

### Database

**Figure 5:**
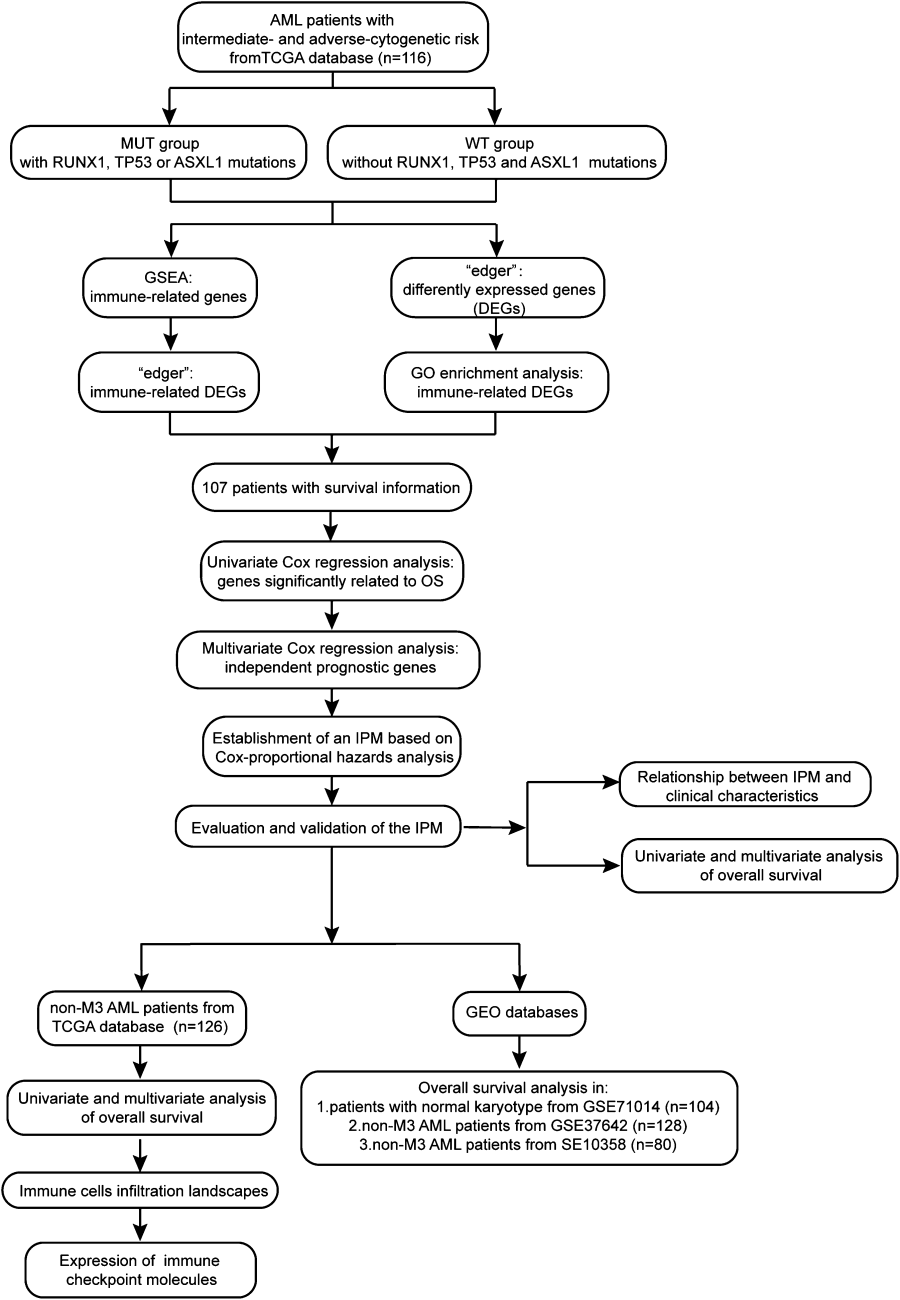
Workflow chart of data generation and analysis.

The workflow is shown in Fig. 5. The somatic mutation status (workflow type: VarScan2 Variant Aggregation and Masking), transcriptional profiles, and the corresponding clinical and overall survival (OS) data of 173 AML patients were downloaded from The Cancer Genome Atlas (TCGA) database (https://portal.gdc.cancer.gov/). The gene expression profile was measured experimentally using the Illumina HiSeq 2000 RNA Sequencing platform. The gene symbols were annotated based on Homo sapiens GRCh38.91.chr.gtf file (http://asia.ensembl.org/index.html). Log2 transformations were performed for all gene expression data. The study reported herein fully satisfies the TCGA publication requirements (http://cancergenome.nih.gov/publications/publicationguidelines). The definition of cytogenetic risk and risk related somatic mutations were based on NCCN guidelines^7^. Of 173 AML patients in TCGA database, 116 patients with intermediate and adverse-cytogenetic risk had available gene expression profile and mutation status while 107 patients had survival information..

To validate the predictive ability of the IPM based on TCGA data, 3 GEO cohorts with survival information were used, they are GSE71014 including 104 AML patients with normal karyotype (based on GPL10558 Illumina HumanHT-12 V4.0 expression beadchip), GSE37642 including 128 non-M3 AML patients and GSE10358 including 80 non-M3 AML patients (based on GPL570 Affymetrix Human Genome U133 Plus 2.0 Array), and the individual clinical and OS information were downloaded from the GEO database (https://www.ncbi.nlm.nih.gov/geo/).

### Identification of differentially expressed genes

First, the raw counts of gene expression data from TCGA were normalized using a weighted trimmed mean of log ratios-based method^24^. To obtain differentially expressed genes (DEGs) between patients with *RUNX1*, *TP53*, or *ASXL1* mutations (MUT group) and without *RUNX1*, *TP53*, and *ASXL1* mutations (WT group), the “edger” package in R software (Version 3.6.2; https://www.r-project.org/) was used. |log2FC|≥ 1.0 and FDR < 0.05 were selected as DEGs.

### Functional enrichment analysis of the DEGs

Functional enrichment analysis of DEGs between the MUT and WT groups was performed based on clusterProfiler, enrichplot, and org.Hs.eg.db packages to identify the immune-related DEGs involved in the immune-related biological processes (BP) of Gene Ontology (GO) categories. FDR < 0.05 was considered statistically significant.

### Gene set enrichment analysis (GSEA)

We used TCGA genomewide expression profiles and selected an annotated gene set file (c5.bp.v6.2.symbols.gm) as the reference gene set to perform GSEA (Version 3.0; http://software.broadinstitute.org/gsea/index.jsp) analysis for identifying immunological pathways and corresponding immune-related genes differ between the MUT and WT groups^23^. The GSEA threshold for significantly enriched immune-related functional annotations was set at *P* < 0.05, false discovery rate (FDR) < 0.25, and a normalized enrichment score > 1.5. Likely, the “edger” package in R software with criteria of |log2FC|≥ 1.0 and FDR < 0.05 was also used to identify the immune-related DEGs in immune-related genes obtained from GSEA.

### Establishment and validation of an immune prognostic model

Univariate Cox regression analysis was performed using the R package “survival” to evaluate correlations between the DEG expression levels and OS of TCGA AML patients with intermediate and adverse-cytogenetic risk. DEGs with *P* < 0.05 by univariate Cox regression analysis were identified as alternative prognostic genes. Among the immune genes that were significant in the univariate Cox regression analysis, a sub-selection of immune genes involved in prognosis was determined by multivariate Cox regression analysis, and in this analysis, genes were regarded as significant at *P* < 0.05. Finally, an IPM was constructed based on Cox-proportional hazards analysis, and the risk score derived from the IPM was calculated by utilizing the “predict” function in R software to assess each patient’s risk. Patients with available survival data were separated into IPM-defined high-risk (IPM-HR) and IPM-defined low-risk (IPM-LR) groups using the optimal cutoff obtained from a receiver operating characteristic (ROC) curve. The predictability of the IPM was evaluated by area under ROC curve (AUC); the higher the value of the AUC, the better the predictability of the model.

### Estimation of immune cell-type fractions

Cell type identification by estimating relative subsets of RNA transcripts (CIBERSORT) is an approach to characterize the cell composition of complex tissues based on gene expression profiles, and has been demonstrated to be highly consistent with ground truth estimations in many cancers^25^. We uploaded normalized gene expression data with standard annotation files to the CIBERSORT web portal. A leukocyte gene signature matrix termed LM22 was used to distinguish 22 immune cell types, including myeloid subsets, natural killer (NK) cells, plasma cells, naïve and memory B cells, and seven T cell types. We used CIBERSORT in combination with the LM22 signature matrix to estimate the fractions of 22 immune cell types between the IPM-HR and IPM-LR AML groups. The sum of all estimated immune cell type fractions was 100% for each sample.

### Statistical analysis

Pairwise comparisons of the variables between groups were performed using the Mann–Whitney U test for continuous variables and Fisher's exact test for categorical variables. Survival functions were estimated using the Kaplan–Meier method and compared using the log-rank test. Variables associated with *P* ≤ 0.25 in the univariate analysis were entered into a multivariate analysis. Comparisons of immune cell type fractions and checkpoint molecules between the IPM-HR and IPM-LR groups were performed using the Mann–Whitney *U* test. The level for a statistically significant difference was set at *P* < 0.05. The SPSS 22.0 software package (SPSS Inc., Chicago, IL) and GraphPad Prism 5 (GraphPad Software Inc., La Jolla, CA) were used for data analysis.

## Supplementary Information

Supplementary Information
