# Supplementary material for: Development of a poor-prognostic-mutations derived immune prognostic model for acute myeloid leukemia
Source: Sci Rep. 2021 Mar 1;11:4856. doi: 10.1038/s41598-021-84190-0 (PMC7921432; doi:10.1038/s41598-021-84190-0)
Supplement: Supplementary file 1 — Supplementary Information [file 41598_2021_84190_MOESM1_ESM.docx]

Development of a poor prognostic mutations-derived immune prognostic model for acute myeloid leukemia

**Authors**

Feng-Ting Dao^#^, Jun Wang^#^, Lu Yang, Ya-Zhen Qin^*^

**Institutional affiliations**

Peking University People's Hospital, Peking University Institute of Hematology, National Clinical Research Center for Hematologic Disease, Beijing, China

*** Correspondence:**

Ya-Zhen Qin, qin2000@aliyun.com, No. 11 Xizhimen South Street, Xicheng District, Beijing, 100044, China.

*** Co-first author:**

Feng-Ting Dao and Jun-Wang contribute equally to this article

**Table S1**. Univariate Cox regression analysis of 64 immune-related DEGs associated with RUNX1, TP53 and ASXL1 mutations in AML patients with intermediate and adverse-cytogenetic risk in the TCGA cohort

| Gene | HR | P value |
| --- | --- | --- |
| PEAR1 | 1.18 | 0.0065 |
| PYCARD | 1.42 | 0.0092 |
| PTK2 | 1.19 | 0.012 |
| IGHV4.59 | 0.88 | 0.062 |
| CD226 | 1.25 | 0.066 |
| CMKLR1 | 1.17 | 0.075 |
| IL7 | 1.16 | 0.079 |
| ROBO1 | 1.20 | 0.079 |
| C2 | 1.12 | 0.10 |
| BLNK | 1.10 | 0.11 |
| SKAP1 | 1.15 | 0.11 |
| PLCG1 | 1.15 | 0.13 |
| IGHJ1 | 0.90 | 0.14 |
| CSF1 | 1.14 | 0.15 |
| ARTN | 1.11 | 0.15 |
| CD22 | 1.15 | 0.16 |
| PLXNA4 | 1.18 | 0.16 |
| LILRA4 | 1.10 | 0.17 |
| SEMA3C | 1.15 | 0.17 |
| IGHG2 | 0.92 | 0.17 |
| RARRES2 | 0.83 | 0.18 |
| CD274 | 1.22 | 0.18 |
| IGHV3.21 | 0.92 | 0.20 |
| IGKC | 0.92 | 0.20 |
| PDGFB | 1.21 | 0.20 |
| IGHV3.43 | 0.92 | 0.22 |
| CARD11 | 1.11 | 0.23 |
| CCL1 | 0.92 | 0.27 |
| IGHV3.30 | 0.93 | 0.27 |
| IGHV3.13 | 0.92 | 0.27 |
| HFE | 1.18 | 0.27 |
| IGHM | 1.08 | 0.32 |
| OSMR | 0.84 | 0.34 |
| C7 | 0.91 | 0.37 |
| TNFRSF21 | 1.07 | 0.39 |
| LBP | 0.93 | 0.41 |
| DEFB1 | 0.96 | 0.41 |
| LGR6 | 1.08 | 0.41 |
| FCER1A | 1.04 | 0.45 |
| TRAV21 | 1.08 | 0.47 |
| C3AR1 | 1.05 | 0.47 |
| BACH2 | 1.08 | 0.47 |
| PTGER3 | 1.08 | 0.48 |
| OTUD7B | 1.14 | 0.49 |
| CR1L | 0.93 | 0.51 |
| CLEC4C | 1.04 | 0.52 |
| UBASH3A | 1.07 | 0.54 |
| CR2 | 1.07 | 0.59 |
| MNX1 | 0.93 | 0.68 |
| CFD | 0.98 | 0.68 |
| IRF4 | 1.04 | 0.71 |
| AZU1 | 0.99 | 0.74 |
| SEMA6C | 1.04 | 0.74 |
| IGHD | 0.98 | 0.74 |
| RNASE3 | 0.99 | 0.81 |
| SLAMF7 | 0.98 | 0.82 |
| IGKV2.28 | 0.98 | 0.86 |
| PDE4B | 1.02 | 0.87 |
| IGLJ1 | 1.01 | 0.91 |
| THY1 | 1.02 | 0.91 |
| CD40LG | 1.01 | 0.93 |
| PROS1 | 1.01 | 0.94 |
| C1S | 1.01 | 0.96 |
| ANXA1 | 1.0 | 0.97 |

**Table S2**. Univariate analysis of OS in AML patients with intermediate and adverse-cytogenetic risk in the TCGA cohort

| Variable | HR(95%CI) | P value |
| --- | --- | --- |
| IPM-HR | 3.74 (2.16-6.50) | < 0.0001 |
| RUNX1mutation (+) | 1.50 (0.80-2.80) | 0.21 |
| TP53 mutation (+) | 3.98 (1.98-7.98) | < 0.0001 |
| ASXL1 mutation (+) | 1.37 (0.34-5.62) | 0.66 |
| FLT3-ITD (+) | 1.42 (0.80-2.52) | 0.23 |
| NPM1 mutation (+) | 0.91 (0.56-1.47) | 0.69 |
| WT1 mutation (+) | 0.79 (0.32-1.96) | 0.61 |
| DNMT3A mutation (+) | 1.70 (1.04-2.78) | 0.035 |
| CEBPA biallelic mutation (+) | 0.39 (0.054-2.79) | 0.35 |
| Age ≥ 60y | 2.39 (1.50-3.83) | < 0.0001 |
| Male | 1.08 (0.68-1.70) | 0.75 |
| WBC > 10 × 10^9^/L | 0.95 (0.60-1.50) | 0.83 |
| Hemoglobin < 90g/L | 1.08 (0.63-1.86) | 0.78 |
| Platelet count < 10 × 10^9^/L | 0.97 (0.57-1.65) | 0.92 |
| BM blast percentage > 35% | 0.81 (0.51-1.27) | 0.35 |
| Adverse-cytogenetic risk | 1.44 (0.88-2.37) | 0.15 |

**Table S3.** Relationship between IPM-defined risk and patient characteristics at diagnosis in AML patients with intermediate and adverse-cytogenetic risk in the TCGA cohort

| Variable | All | IPM defined risk | | P value |
| --- | --- | --- | --- | --- |
|  |  | IPM-LR | IPM-HR |  |
| Number of patients | 107 | 87 | 20 |  |
| Age (year, median, range) | 60 (21-88) | 56 (21-81) | 70 (43-88) | <0.0001 |
| Males (%) | 61 (57.0%) | 50 (57.5%) | 11 (55.0%) | 0.84 |
| WBC (× 109/L, median, range) | 16.0 (1.0-224.0) | 17.0 (1.0-224.0) | 15.5 (2.0-172.0) | 0.81 |
| Hemoglobin (g/L) | 90.0 (60.0-130.0) | 90.0 (60.0-130.0) | 90.0 (80.0-130.0) | 0.94 |
| Platelet count (× 109/L, median, range) | 50.0 (9.0-351.0) | 45.0 (9.0-351.0) | 62.5 (19.0-166.0) | 0.35 |
| BM blast (%, median, range) | 35% (0-97%) | 35% (0-97%) | 33% (0-91%) | 0.76 |
| FAB classification, n (%) |  |  |  | 0.59 |
| M0 | 14 | 9 (16.9%) | 5 (0) |  |
| M1 | 27 | 22 (26.5%) | 5 (20.8%) |  |
| M2 | 27 | 24 (30.1%) | 3 (8.3%) |  |
| M3 | 1 | 1 (1.2%) | 0 (0) |  |
| M4 | 20 | 16 (18.1%) | 4 (20.8%) |  |
| M5 | 14 | 12 (3.6%) | 2 (45.8%) |  |
| M6 | 2 | 1 (2.4%) | 1 (0) |  |
| M7 | 1 | 1 (1.2%) | 0 (0) |  |
| Karyotype, n (%) |  |  |  |  |
| Normal karyotype | 63 (58.9%) | 52 (59.8%) | 11 (55.0%) | 0.79 |
| others | 34 (31.8%) | 27 (31.0%) | 7 (35.0%) |  |
| Cytogenetics risk category, n (%) |  |  |  |  |
| Intermediate | 75 (70.1%) | 63 (72.4%) | 12 (60.0%) | 0.29 |
| Poor | 32 (29.9%) | 24 (27.6%) | 8 (40.0%) |  |
| RUNX1, n (%) |  |  |  |  |
| Mutation | 14 (13.1%) | 8 (9.2%) | 6 (30.0%) | 0.023 |
| Wild type | 93 (86.9%) | 79 (90.8%) | 14 (70.0%) |  |
| ASXL1, n (%) |  |  |  |  |
| Mutation | 2 (1.9%) | 1 (1.2%) | 1 (5.0%) | 0.34 |
| Wild type | 105 (98.1%) | 86 (98.8%) | 19 (95.0%) |  |
| TP53, n (%) |  |  |  |  |
| Mutation | 10 (9.3%) | 7 (8.1%) | 3 (15.0%) | 0.39 |
| Wild type | 97 (90.7%) | 80 (91.9%) | 17 (85.0%) |  |
| NPM1, n (%) |  |  |  |  |
| Mutation | 35 (32.7%) | 32 (36.8%) | 3 (15.0%) | 0.069 |
| Wild type | 72 (67.3%) | 55 (63.2%) | 17 (85.0%) |  |
| FLT3-ITD, n (%) |  |  |  |  |
| (+) | 21 (19.6%) | 19 (21.8%) | 2 (10.0%) | 0.35 |
| (-) | 86 (80.4%) | 68 (78.2%) | 18 (90.0%) |  |
| CEBPA, n (%) |  |  |  |  |
| Biallelic mutation | 4 (3.7%) | 4 (4.6%) | 0 (0) | 0.33 |
| Wild type | 103 (96.3%) | 83 (95.4%) | 20 (100%) |  |
| WT1, n (%) |  |  |  |  |
| Mutation | 8 (7.5%) | 8 (9.2%) | 0 (0) | 0.35 |
| Wild type | 99 (92.5%) | 79 (90.8%) | 20 (100%) |  |
| DNMT3A, n (%) |  |  |  |  |
| Mutation | 33 (30.8%) | 30 (34.5%) | 3 (15.0%) | 0.11 |
| Wild type | 74 (69.2%) | 57 (65.5%) | 17 (85.0%) |  |

**Table S4**. Univariate analysis of OS in non-M3 AML patients in the TCGA cohort

| Variable | HR(95%CI) | P value |
| --- | --- | --- |
| IPM-HR | 4.19 (2.47-7.10) | < 0.0001 |
| RUNX1mutation (+) | 1.65 (0.89-3.07) | 0.11 |
| TP53 mutation (+) | 4.34 (2.17-8.68) | < 0.0001 |
| ASXL1 mutation (+) | 1.52 (0.37-6.21) | 0.56 |
| FLT3-ITD (+) | 1.55 (0.90-2.66) | 0.12 |
| NPM1 mutation (+) | 1.12 (0.70-1.78) | 0.63 |
| WT1 mutation (+) | 0.83 (0.36-1.91) | 0.66 |
| DNMT3A mutation (+) | 1.96 (1.21-3.16) | 0.006 |
| CEBPA biallelic mutation (+) | 0.55 (0.14-2.24) | 0.40 |
| Age ≥ 60y | 2.64 (1.70-4.12) | < 0.0001 |
| Male | 1.08 (0.70-1.66) | 0.74 |
| WBC > 10 × 10^9^/L | 0.91 (0.59-1.42) | 0.68 |
| Hemoglobin < 90g/L | 1.02 (0.61-1.70) | 0.95 |
| Platelet count < 10 × 10^9^/L | 1.0 (0.60-1.69) | 0.99 |
| BM blast percentage > 35% | 0.91 (0.60-1.40) | 0.68 |
| Intermediate and adverse-cytogenetic risk | 2.84 (1.23-6,25) | 0.014 |

**Table S5**. Clinical characters of GEO cohorts.

| Variables | GSE71014 | | GSE37642 | | GSE10358 | |
| --- | --- | --- | --- | --- | --- | --- |
| Number of patients | 104 | | 128 | | 80 | |
| Age (year, median; range) | UA* | 60 (18-85) | | UA | |  |
| Male (%) | UA | | UA | | 38 (47.5%) | |
| FAB classification, n (%) | UA | |  | |  | |
| M0 |  | | 8 (6.3%) | | 7 (8.7%) | |
| M1 |  | | 29 (22.6%) | | 21 (26.3%) | |
| M2 |  | | 47 (36.7%) | | 22 (27.5%) | |
| M4 |  | | 17 (13.3%) | | 17 (21.3%) | |
| M5 |  | | 19 (14.8%) | | 12 (15.0%) | |
| M6 |  | | 7 (5.5%) | | 0 (0) | |
| M7 |  | | 1 (0.8%) | | 1 (1.2%) | |

*UA means these clinical characters of cohorts were unavailable.
